# Enhancing the hypotensive effect of latanoprost by combining synthetic phosphatidylcholine liposomes with hyaluronic acid and osmoprotective agents

**DOI:** 10.1007/s13346-024-01584-z

**Published:** 2024-04-11

**Authors:** Marco Brugnera, Marta Vicario-de-la-Torre, Miriam Ana González-Cela Casamayor, José Javier López-Cano, Irene Bravo-Osuna, Fernando Huete-Toral, María Luisa González Rubio, Gonzalo Carracedo, Irene Teresa Molina-Martínez, Vanessa Andrés-Guerrero, Rocío Herrero-Vanrell

**Affiliations:** 1 Innovation, Therapy and Pharmaceutical Development in Ophthalmology (InnOftal) Research Group, Complutense University of Madrid (UCM), Madrid, Spain; 2 Department of Pharmaceutics and Food Technology, Faculty of Pharmacy, UCM; IdISSC, Madrid, Spain; 3 University Institute of Industrial Pharmacy (IUFI), Faculty of Pharmacy, UCM, Madrid, Spain; 4 Ocupharm Research Group, Department of Optometry and Vision, Faculty of Optics and Optometry, UCM, Madrid, Spain

**Keywords:** Glaucoma, Liposomes, Latanoprost, Hyaluronic acid, Osmoprotectants, Ocular drug delivery

## Abstract

The first line of glaucoma treatment focuses on reducing intraocular pressure (IOP) through the prescription of topical prostaglandin analogues, such as latanoprost (LAT). Topical ophthalmic medicines have low bioavailability due to their rapid elimination from the ocular surface. Nanotechnology offers innovative ways of enhancing the ocular bioavailability of antiglaucoma agents while reducing administration frequency. This study aims to combine LAT-loaded synthetic phosphatidylcholine liposomes with hyaluronic acid (0.2% *w/v*) and the osmoprotectants betaine (0.40% *w/v*) and leucine (0.90% *w/v*) (LAT-HA-LIP) to extend the hypotensive effect of LAT while protecting the ocular surface. LAT-HA-LIP was prepared as a mixture of 1,2-dioleoyl-sn-glycero-3-phosphocholine and 1,2-dimyristoyl-sn-glycero-3-phosphocholine, cholesterol and α-tocopherol acetate. LAT-HA-LIP exhibited high drug-loading capacity (104.52 ± 4.10%), unimodal vesicle sizes (195.14 ± 14.34 nm) and a zeta potential of -13.96 ± 0.78 mV. LAT-HA-LIP was isotonic (284.00 ± 1.41 mOsm L^−1^), had neutral pH (7.63 ± 0.01) and had suitable surface tension (44.07 ± 2.70 mN m^−1^) and viscosity (2.69 ± 0.15 mPa s^−1^) for topical ophthalmic administration. LAT-HA-LIP exhibited optimal in vitro tolerance in human corneal and conjunctival epithelial cells. No signs of ocular alteration or discomfort were observed when LAT-HA-LIP was instilled in albino male New Zealand rabbits. Hypotensive studies revealed that, after a single eye drop, the effect of LAT-HA-LIP lasted 24 h longer than that of a marketed formulation and that relative ocular bioavailability was almost three times higher (p < 0.001). These findings indicate the potential ocular protection and hypotensive effect LAT-HA-LIP offers in glaucoma treatment.

**Graphical abstract:**

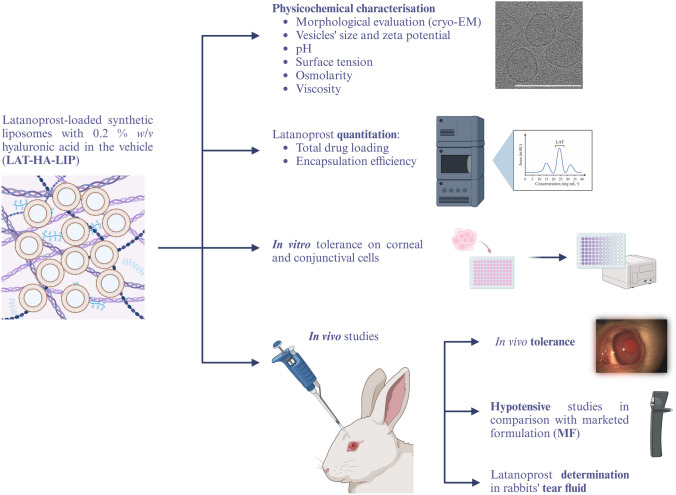

**Supplementary Information:**

The online version contains supplementary material available at 10.1007/s13346-024-01584-z.

## Introduction

Glaucoma is the most common cause of permanent blindness today. Experts estimate that within 20 years’ time approximately 111.8 million people will be affected by glaucoma [1, 2]. This disease is characterised by chronic and multifactorial features and its pathogenesis is still being investigated [3]. Both glaucoma onset and progression are characterised by several risk factors, the main one being an increase in intraocular pressure (IOP), which is triggered by impaired aqueous humour dynamics. When high IOP persists over time, glaucoma patients may undergo progressive retinal ganglion cell and optic nerve degeneration and, subsequently, irreversible vision loss [4]. Currently, IOP reduction is used to prevent progression of the disease, either by decreasing the production of aqueous humour, improving its drainage efficiency or managing episcleral vein pressure [5].

Amongst the therapeutic drugs currently employed to reduce IOP, topical prostaglandin analogues such as bimatoprost, travoprost, tafluprost or latanoprost represent the first line of glaucoma treatment alongside α-2 adrenergic agonists, β-blockers and carbonic anhydrase inhibitors. Prostaglandin analogues increase aqueous humour outflow by relaxing ciliary smooth muscles, changing the cytoskeleton and altering the extracellular matrix of the uveoscleral pathway [6]. Latanoprost (LAT), in particular, acts as a selective agonist at the endogenous prostaglandin F_2α_ receptor. It is a prodrug that has to be hydrolysed by ocular esterases in latanoprost acid to be therapeutically active when it reaches the aqueous humour [7]. LAT is currently considered one of the most effective drugs for treating glaucoma [8, 9]. At present, several topical LAT medications are commercially available in different presentations (single-dose, multi-dose and preservative-free multi-dose eyedropper) in solution or emulsion form, at 0.005% *w/v* [10–12].

Despite their convenient and non-invasive nature, topical ophthalmic medicines have poor bioavailability due to their rapid elimination from the ocular surface, the low permeability of the corneal epithelium to active substances, their non-desired absorption in the conjunctiva, and nasolacrimal drainage. Furthermore, chronic topical administration of formulations may alter the homoeostasis of the ocular surface, thus destabilising the preocular tear film. Its disruption can cause tear hypertonicity and ocular surface desiccation. Additionally, the hyperosmolar environment can activate apoptosis in ocular surface epithelial cells, triggering dry eye disease (DED) [13]. Development of DED is directly linked to lack of patient compliance, which can lead to non-optimal IOP values, thus compromising the efficacy of the treatment, and may, subsequently, raise healthcare expenses. The adverse effects caused on the ocular surface by topical glaucoma medications are mainly associated with the nature of the preservative included in the eye drops. The hallmark for decelerating the strong correlation between DED emergence and glaucoma treatment has been the use of unpreserved hypotensive medications to minimise ocular surface inflammatory response [14]. However, withholding preservatives from formulations is not enough to prevent toxicity in chronic treatments since hypotensive active substances can also damage the ocular surface, as is the case with prostaglandin analogues. For these reasons, novel strategies are needed to increase the bioavailability of these antiglaucoma drugs without adversely affecting the ocular surface.

Nanotechnology can play a part in enhancing drug bioavailability and increasing ocular compatibility [15–18]. Liposomes have been widely employed as biodegradable and biocompatible drug delivery systems for ophthalmic administration. They have gained attention over the years due to their ability to improve the bioavailability of poorly water-soluble compounds and so enhance cellular uptake, reduce potential toxicity and increase ocular barrier penetration. Encapsulating prostaglandin analogues in liposomes provides protection from esterase activity in the physiological environment, and particularly at the corneal epithelium interface, thus enhancing their stability and improving their bioavailability [19, 20]. Liposomes are composed of an aqueous core surrounded by one or more lipid bilayers. The latter are generally composed of phospholipids (one of the main components present in the natural tear film), which have already been used in artificial tear compositions to restore the lipid tear film layer and subsequently slow tear film water loss produced by evaporation [21]. In this paper, a mixture of saturated and unsaturated synthetic phosphatidylcholine derivatives is used as the main component of the nanovesicles due to the latter's advantages over natural phospholipids in terms of standardisation, characterisation and potential industrial-scale liposome production [22].

In addition to including a hypotensive drug in liposomes, another useful strategy to increase eye drop bioavailability and stability is to add a mucoadhesive polymer to the composition [23, 24]. To this end, hyaluronic acid (HA) has been incorporated in the formulation vehicle due to its tuneable viscosity and mucoadhesive capabilities. HA is a natural polysaccharide found in the tear film and vitreous humour. It is a biocompatible and biodegradable polymer that provides long-lasting hydration at the ocular surface [25]. Its mucoadhesive properties originate from the hydrogen bonds established with the tear film's aqueous/mucin layer and the glycocalyx coating on the apical corneal epithelial cells, which improve the ocular residence time of HA-based medications [26, 27]. Given its distinctive characteristics, it is no wonder that HA is frequently employed in artificial tears to treat DED [28, 29].

Additionally, tear evaporation and the consequent formation of a hyperosmolar environment can activate apoptosis in ocular surface epithelial cells and further disrupt tear film stability. This phenomenon can be thwarted by adding osmoprotective substances (e.g. compounds able to modify cellular water uptake and protect the ocular surface from desiccation) to artificial tears. Osmoprotectants have demonstrated their capacity to prevent and decelerate the initiation of DED and can additionally be utilised after manifestation of damage [30]. In this study, betaine (BET) and leucine (LEU) were the two osmoprotectants included in the ophthalmic formulations. The capacity of BET to safeguard ocular cells exposed to evaporative and hypertonic stress and to reduce apoptosis has been demonstrated [31]. Meanwhile, LEU, one of the predominant amino acids within the collagen I and corneal stroma, exhibits anti-inflammatory properties. Moreover, both these substances are also able to decrease inflammation and oxidative processes linked to DED [32–34]. Osmoprotective substances have been already combined with HA in artificial tears to provide relief and hydration and, most importantly, to act synergistically in the treatment of signs and symptoms of DED [35, 36].

The aim of this research is therefore to evaluate the combination of two technological resources capable of increasing the ocular bioavailability of LAT and to protect the ocular surface against the adverse effects of its chronic application. The first aim is to include the active ingredient in the synthetic liposomes’ phospholipid bilayer as a nanosized carrier. The addition of HA, meanwhile, is intended to extend the formulation’s ocular residence time. Moreover, osmoprotective substances (BET and LEU) were added during liposome preparation to prevent damage to the ocular surface and the consequent development of DED. The liposomal formulations prepared were physicochemically evaluated in terms of morphology, size, pH, surface tension, osmolarity and viscosity. To study in vitro tolerance and in vivo safety and efficacy, Monoprost^®^, a commercially available preservative-free sterile 0.005% *w/v* LAT-loaded single-dose ophthalmic solution (MF), was used as a benchmark. To authors’ knowledge, there is no liposome-based formulation for glaucoma treatment on the market.

## Materials and methods

### Chemicals

1,2-dioleoyl-sn-glycero-3-phosphocholine (DOPC) (LIPOID PC 18:1/18:1, batch 556890-216 01/073, CAS 4235-95-4) and 1,2-dimyristoyl-sn-glycero-3-phosphocholine (DMPC) (LIPOID PC 14:0/14:0, batch 562236-01/028, CAS 18194-24-6) were purchased from Lipoid GmbH (Ludwigshafen, Germany). Latanoprost (LAT) (HY-B0577/CS-2758, batch 90,610, CAS 130203-82-4) was obtained from MedChemExpress (Monmouth Junction, New Jersey, United States of America). Cholesterol (CHOL) (C8667-5G, batch SLBW6939, CAS 57-88-5, ≥ 99%), α-tocopherol acetate (VE) (T3376-5G, batch MKCM6113, CAS 7695-91-2, ≥ 96%), L-leucine (LEU) (L8000-100G, batch BCBZ3028, CAS 61-90-5, ≥ 98%), trifluoroacetic acid (TFA) (302031-100ML, batch 102532920, CAS 76-05-1, ≥ 99%), 3-(4,5-dimethylthiazol-2-yl)-2,5-diphenyltetrazolium bromide (MTT) (475989, CAS 298-93-1, ≥ 98%), benzalkonium chloride (BAK) (B6295, batch 1298263, CAS 63449-41-2), boric acid (H_3_BO_3_) (B6768-1 KG, batch BCBS7652, CAS 10043-35-3, ≥ 99.5%) and sodium tetraborate decahydrate (Na_2_B_4_O_7_·10H_2_O) (S9640-2.5 KG, batch BCCB7490, CAS 1303-96-4, ≥ 99.5%) were supplied by Sigma-Aldrich (Madrid, Spain). Sodium chloride (NaCl) was obtained from Merck (1.06404.1000, Merck KGaA, Darmstadt, Germany). Betaine anhydrous (BET) (204241000, batch A0419439, CAS 107-43-7, ≥ 98%) and D-(+)-trehalose dihydrate (TREH) (BP2687-100, batch 215417, CAS 6138-23-4) were purchased from Fisher Scientific (Geel, Belgium). Acetonitrile (221881.1612) and dimethyl sulfoxide (DMSO) (A3672,0100) were acquired from PanReac AppliChem (Barcelona, Spain). Ophthalmic-grade sodium hyaluronate (HA) (F002503, batch 6/0001, molecular weight 400–800 kDa), was supplied by Abaran Materias Primas S.L. (Madrid, Spain). Single-dose Monoprost^®^ (MF) (Laboratoires Théa, Madrid, Spain) and single-dose Lusan^®^ (0.9% *w/v* NaCl) (Hartmann SA, Barcelona, Spain) were used as benchmarks for the in vivo studies. Water was purified using a Milli-Q^®^ filtration system (Millipore Corporation, Billerica, MA, USA).

### Liposome manufacture

**Fig. 1:**
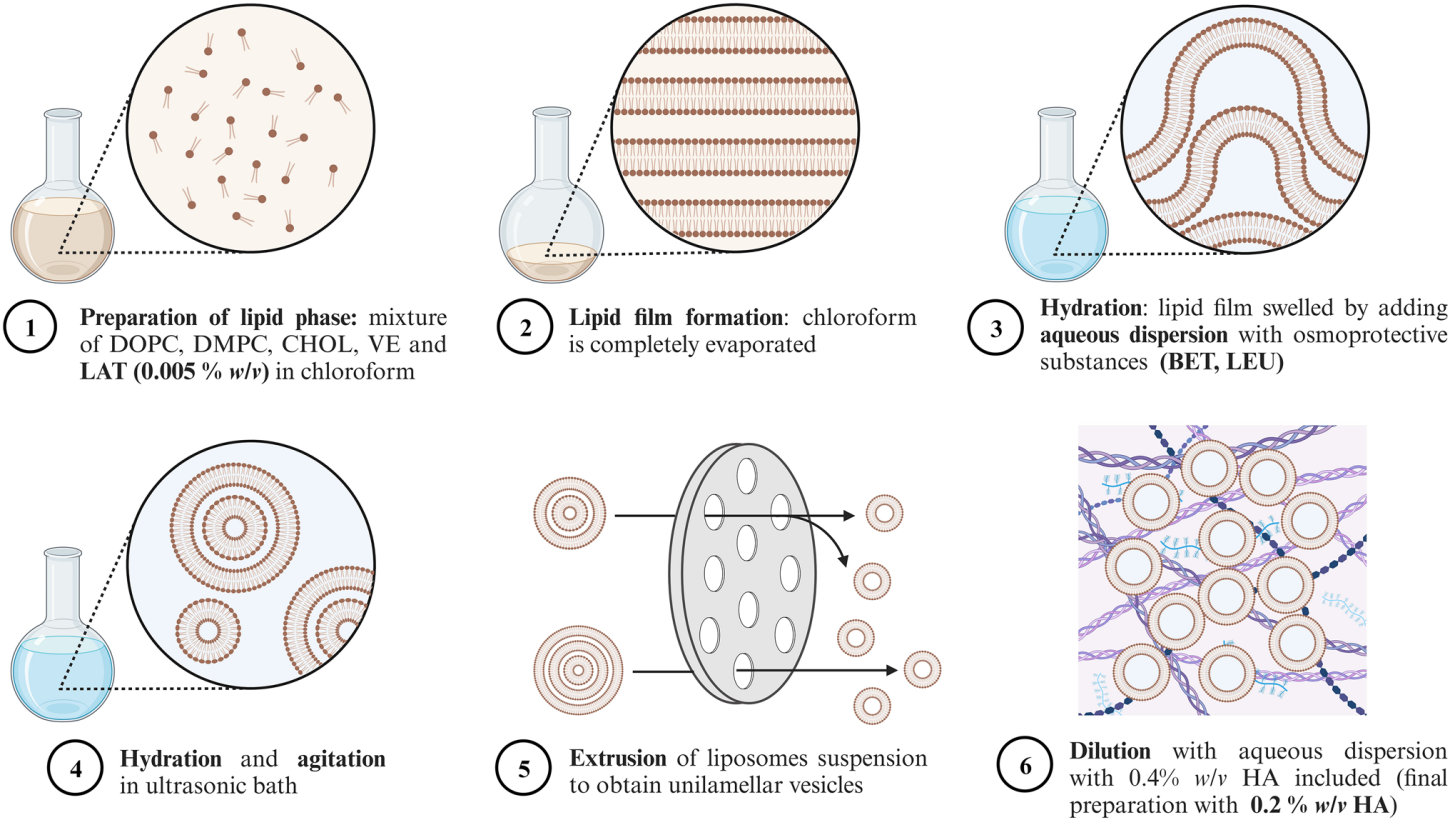
Detailed diagram of liposome manufacture as per the lipid film hydration method. The image was created with BioRender (created on 13 November 2023)

Liposomes were prepared as per the lipid film hydration protocol developed by Bangham et al., albeit with some modifications, as shown in Fig. 1 [37, 38]. Components were selected considering the mechanical characteristics and in vitro tolerance results reported in previous studies [34, 38, 39]. The aqueous phase was composed of a borate buffer (0.84% *w/v* H_3_BO_3_, 0.14% *w/v* Na_2_B_4_O_7_·10H_2_O), 1.04% *w/v* TREH, 0.40% *w/v* BET and 0.90% *w/v* LEU. DOPC, DMPC, CHOL and VE were respectively incorporated in a weight ratio of 6:2:1:0.08 to constitute the lipid phase. First, the aforementioned lipid mixture was dissolved in chloroform and then placed in a rotary evaporator (Buchi R-205, Massó Analítica S.A., Spain) at reduced pressure (100 mPa and 50 mPa for 30 min, at 150 rpm) at 33 °C. Once this step was completed, organic solvent traces were removed with nitrogen flow for 30 s. Next, 10 mL of the aqueous phase were added to swell the lipid film at 185 rpm for 15 min. The liposomes obtained were left to stand at room temperature for 2 h and were then subjected to sonication in an ultrasonic bath for 15 min. (Bandelin^®^ Sonorex Digiplus, DL 510 H, Berlin, Germany). Unilamellar vesicles were obtained with a high-pressure extruder (Lipex Biomembrane™, Vancouver, BC, Canada) by passing the liposomes through a 0.8 µm membrane (Nuclepore™ Track-Etch Membrane, 10417304, batch A30003737, Whatman™, Cytiva Europe GmbH, Freiburg, Germany) for 10 cycles, then by passing them through 0.2 µm polycarbonate filters (Nuclepore™ Track-Etch Membrane, 110606, batch 7084288, Whatman™, Cytiva Europe GmbH, Freiburg, Germany) for 10 cycles at 25 °C under a fume hood. At the end of the extrusion process the liposomes were diluted 1:1 with the aqueous phase (the same phase mentioned above) to achieve the desired final phospholipid concentration (1% *w/v*). To obtain liposomes dispersed in 0.2% *w/v* HA, 0.4% *w/v* HA was added to the aqueous phase beforehand and was then used to dilute (1:1) the liposomes after the extrusion cycles. After dilution, the liposomes were left overnight at 4 °C to allow full hydration.

In the case of the LAT-loaded liposomes, LAT dissolved in chloroform was included in the lipid phase to achieve a final concentration of 50 µg mL^−1^. The liposomal formulations produced, their lipid bilayer and their aqueous phase compositions are detailed in Table 1 below.

**Table 1:** Composition of the liposomal formulations: unloaded synthetic liposomes (B-LIP), unloaded synthetic liposomes with 0.2% w/v HA (B-HA-LIP), LAT-loaded synthetic liposomes (LAT-LIP) and LAT-loaded synthetic liposomes with 0.2% w/v HA (LAT-HA-LIP)

Liposomal formulation	Lipid bilayer	Aqueous phase
B-LIP	DOPC = 0.75%* w/v* DMPC = 0.25%* w/v* CHOL = 0.125%* w/v* VE = 0.01%* w/v*	H_3_BO_3_ = 0.84%* w/v* Na_2_B_4_O_7_·10H_2_O = 0.14%* w/v* TREH = 1.04%* w/v* BET = 0.40%* w/v* LEU = 0.90%* w/v*
B-HA-LIP	DOPC = 0.75%* w/v* DMPC = 0.25%* w/v* CHOL = 0.125%* w/v* VE = 0.01%* w/v*	H_3_BO_3_ = 0.84%* w/v* Na_2_B_4_O_7_·10H_2_O = 0.14%* w/v* TREH = 1.04%* w/v* BET = 0.40%* w/v* LEU = 0.90%* w/v* HA = 0.2%* w/v*
LAT-LIP	DOPC = 0.75%* w/v* DMPC = 0.25%* w/v* CHOL = 0.125%* w/v* VE = 0.01%* w/v* LAT = 0.005%* w/v*	H_3_BO_3_ = 0.84%* w/v* Na_2_B_4_O_7_·10H_2_O = 0.14%* w/v* TREH = 1.04%* w/v* BET = 0.40%* w/v* LEU = 0.90%* w/v*
LAT-HA-LIP	DOPC = 0.75%* w/v* DMPC = 0.25%* w/v* CHOL = 0.125%* w/v* VE = 0.01%* w/v* LAT = 0.005%* w/v*	H_3_BO_3_ = 0.84%* w/v* Na_2_B_4_O_7_·10H_2_O = 0.14%* w/v* TREH = 1.04%* w/v* BET = 0.40%* w/v* LEU = 0.90%* w/v* HA = 0.2%* w/v*

### Physicochemical characterisation of the liposomes

#### Morphological evaluation

The liposomes were visualised using cryo-transmission electron microscopy (cryo-EM) to confirm their formation and explore their structure. The morphology and appearance of the four formulations were evaluated using a 200 kV FEI Talos Arctica device (FEI Company, Hillsboro, Oregon, USA). Regarding the cryo-EM analyses, B-LIP was previously diluted at a ratio of 1:10 and a small amount (3 µL) was subsequently placed on the upper side of Quantifoil^®^ Lacey Carbon support (Cu/Rh lacey carbon grids) and then blotted. An FEI Vitrobot Mark IV device was employed to plunge the sample into liquid ethane. B-LIP was also processed in a Talos Arctica device using an X-field emission gun operating at 200 kV. EPU Software (ThermoFisher Scientific^®^) on a Falcon III device was used to capture the images. These were recorded under low-dose conditions at nominal magnifications of 22,000 (0.94 nm/pixel sampling rate) and 92,000 (0.11 nm/pixel sampling rate) for field and individual captures, respectively. Lastly, the ImageJ software (Fiji, version 1.54d) was used to process each image. The same procedure was repeated for B-HA-LIP, LAT-LIP and LAT-HA-LIP.

#### Vesicle size and zeta potential

Liposome sizes and zeta potential were determined at 25 °C by diluting the samples in Milli-Q^®^ water at a ratio of 1:10. Both parameters were evaluated, respectively, in disposable transparent polystyrene cuvettes (batch 67.754, Sarstedt, Nümbrecht, Germany) and disposable folded capillary cells (DTS1070, Zetasizer Nano Series^®^, Malvern Panalytical Ltd, Malvern, United Kingdom) using the ZS Xplorer^®^ software and Zetasizer Lab^®^ (Malvern Panalytical Ltd, Malvern, United Kingdom). Material refractive index and absorption were correspondingly set at 1.35 and 0.001, whereas water refractive index, viscosity and dielectric constant were respectively set at 1.33, 1.05 and 78.5.

#### pH

The pH of the four different batches was measured in triplicate using a pH meter (model GLP-2, Crison Instruments SA, Barcelona, Spain) calibrated with standards at pH equal to 7.00 and 9.00 and equipped with a microelectrode (InLab, Mettler, Barcelona, Spain).

#### Surface tension

Liposomal formulations surface tension was measured as per the Wilhelmy plate method. Briefly, all measurements were taken in triplicate using a K-11 digital tensiometer (Kruss GmbH, Hamburg, Germany) previously calibrated with Milli-Q^®^ water (70.0 ± 2.0 mN/m) at 33 °C to simulate ocular surface temperature [40]. Before each analysis, the liposomal formulations were pre-heated to 33 °C and equilibrated for 3 min.

#### Osmolarity

Osmolarity was measured by the freezing point depression technique using a Fiske^®^ single-sample micro osmometer (model 210, Fiske^®^ Associates, Norwood, Massachusetts, United States). Three standard solutions of 50, 290 and 850 mOsm/L were employed to calibrate the equipment.

#### Rheological studies

Viscosity was measured by a parallel plate system linked to a Discovery HR1 hybrid rheometer (TA Instruments, New Castle, Delaware, United States). Viscosity was determined by increasing shear rates from 0–1000 s^−1^ in 30 steps. The study was performed at room temperature.

### Quantitation of the latanoprost in the liposomal formulation

High-performance liquid chromatography coupled with ultraviolet detection (HPLC-UV) was employed to quantify the LAT in the formulations produced. The apparatus used to perform the analyses was a Waters^®^ Acquity Arc Bio UHPLC device (Waters, Madrid, Spain) paired with a Waters^®^ Photodiode Array 2998 detector; samples were analysed with the HPLC line of the instrument. The Empower 3^®^ software was used to collect and process the chromatographical results. Analyses were based on a pre-existing method employing an Ascentis^®^ C18 HPLC Column (10 cm × 4.6 mm, 3 µm) (265458-04, Supelco^®^, Sigma-Aldrich, Madrid, Spain) as stationary phase [41]. The reversed-phase HPLC column was kept at 30.0 ± 0.2 °C throughout the analysis. The injection volume was 10 µL and the flow rate was set at 1 mL min^−1^. The composition of the mobile phase was acetonitrile – 0.1% *v/v* TFA in Milli-Q^®^ water (70:30% *v/v*). The isocratic elution was examined for 3.00 min and LAT was detected at 210.0 nm [36]. Several standard dilutions were prepared from a 5 mg mL^−1^ LAT stock solution in acetonitrile (10, 25, 40, 50, 60, 75, 100 µg mL^−1^) to calculate the regression line. The HPLC-UV analyses were only performed in triplicate on the LAT-LIP formulation. Precision, expressed as relative standard deviation percentage, remained lower than 1.55% for all the concentrations of the regression line, whereas the accuracy, expressed as the relative error, was in any case below 1.05%. Furthermore, limit of detection (LOD) and limit of quantitation (LOQ) were considered when LAT was not detectable in the chromatograms in order to determine the maximum range of quantitation. As per IUPAC principles, LOD and LOQ were calculated using the standard error of the intercept (σ) obtained from the calculation of the regression line divided by its slope (m) [42].

$$LOD=\left(\frac{\sigma }{m}\right)\cdot 3.3$$

$$LOQ=(\frac{\sigma }{m})\cdot 10$$

LOD and LOQ resulted respectively equal to 0.27 µg mL^−1^ and 0.83 µg mL^−1^, whereas linearity parameters, σ and m were correspondingly calculated as 899.40 and 10892.12.

#### Total drug loading

To quantify the total LAT loading present (LAT_tot_), the liposomal formulation (1 mL) was lyophilised (Telstar Lyoquest^®^ 30 benchtop freeze dryer, Telstar, Terrassa, Spain) once elaborated (freezing: − 60 °C/60 min, drying: − 60 °C/12 h/0.1 mBar). Afterwards, the same volume of acetonitrile (1 mL) was added to solubilise the pellet. Following centrifugation (5000 rpm, 5 min, 20 °C, Micro 220R, Hettich^®^, Aizarnazabal, Guipuzcoa, Spain) and filtration (0.22 µm-pore nylon syringe filters, JNY022013N, Filter-Lab^®^, Barcelona, Spain), the solution was placed in vials and injected into the chromatographic system as described in 2.4. LAT_tot_ was calculated as the ratio between the LAT concentration present in the analysed samples and the theoretical concentration (50 µg mL^−1^) included in preparation.

$${LAT}_{tot}\, \left[\%\right]=\frac{{[LAT]}_{real} \;in \;LAT-LIP}{{\left[LAT\right]}_{theoretical} \;in\; LAT-LIP}\cdot100$$

#### Encapsulation efficiency

Encapsulation efficiency (EE) refers to the LAT percentage trapped inside the vesicles’ lipid bilayer. Ultrafiltration was employed to determine the LAT concentration present in the aqueous core and in the buffered vehicle, which was then deducted from total LAT concentration [36]. LAT-LIP was diluted with Milli-Q^®^ water at a ratio of 1:10 and a suitable volume (0.5 mL) was pipetted into centrifugal tubes fitted with appropriate filters (Amicon^®^ Ultra 0.5 mL with Ultracel^®^ 50 kDa regenerated cellulose 50000 NMWL, UFC505024, Millipore^®^, Sigma-Aldrich, Madrid, Spain). This was then centrifuged (14000 rpm, 5 min, 20 °C) and filtered. The ultrafiltered solutions were freeze-dried (freezing: − 60 °C/60 min, drying: − 60 °C/12 h/0.1 mBar), incubated in acetonitrile (1 mL) to dissolve the residual LAT present and vortex-mixed (Vortex D-051, Dinko^®^, Barcelona, Spain) for 3 min. Samples were then filtered, put in vials and subjected to HPLC-UV analyses as described in 2.4. Encapsulation efficiency was expressed considering the LOD and LOQ. Considering the total LAT concentration present in the LAT-LIP and the LAT concentration detected after ultrafiltration (LAT_free_), EE was calculated by the following equation:

$$EE \,\left[\%\right]= \frac{{[LAT]}_{tot}- {[LAT]}_{free}}{{[LAT]}_{tot}}\cdot 100$$

### In vitro studies in human corneal and conjunctival cells

Human immortalised corneal epithelial cells (hTERT-HCECs) (Evercyte GmbH, Vienna, Austria) and human immortalised conjunctival epithelial cells (IM-HConEpiC) (Innoprot, Bizkaia, Spain) were used to conduct cytotoxicity studies of the four liposomal formulations, which were previously filtered through 0.22 µm-pore cellulose acetate syringe filters (JCAS022025K, Filter-Lab^®^, Barcelona, Spain). MF was used as the benchmark. This research group has previously investigated the in vitro viability of these liposomal formulations’ components (e.g. synthetic phospholipids and osmoprotective substances, alone and in combination) [38, 43]. Culture cells were maintained under appropriate conditions (37 °C, 5% *v/v* CO_2_, 95% *v/v* humidity) in T75 tissue culture flasks (Sarstedt, Madrid, Spain) and their supplemented media were changed every 2 days. The hTERT-HCECs were cultured with EpiLife™ cell culture medium (Life Technologies Corporation, Madrid, Spain) and supplemented with EpiLife™ Defined Growth Supplement (EDGS^®^ 100X, Life Technologies Corporation, Madrid, Spain) and 1% *w/v* penicillin (10000 units mL^−1^) and streptomycin (10000 µg mL^−1^) (Pen Strep^®^, Life Technologies Corporation, Madrid, Spain). To ensure correct cell attachment and preservation, the flasks were coated with 2% *w/v* gelatine before adding the cell suspension. Conjunctival cells were cultured with IM-Ocular Epithelial Cell Medium (Innoprot, Bizkaia, Spain) and collagen (1 mg mL^−1^) was employed as flask coating (Collagen I Coating Kit, Innoprot, Bizkaia, Spain).

Both hTERT-HCECs and IM-HConEpiC were exposed to the four liposomal formulations for 1 h and 4 h to respectively simulate short-term treatment and chronic topical exposure [44]. The cytotoxicity of the four formulations was evaluated by the MTT assay [38]. hTERT-HCECs and IM-HConEpiC were seeded in 96-well plates (Sarstedt, Madrid, Spain) at a cell density equal to 20000 and 25000 cells per well, respectively, and incubated overnight (16 h). After that, supernatants were discarded and cells were exposed for 1 h and 4 h to B-LIP, B-HA-LIP, LAT-LIP, LAT-HA-LIP ([DOPC-DMPC] = 0.5% *w/v*) and MF using a volume of 100 µL of formulation per well ([LAT] = 0.0025% *w/v*) and 100 µL of the supplemented medium per well. Afterwards, supernatants were carefully removed and MTT solution (0.33 mg mL^−1^) was added to the plates and incubated for 4 h at 37 °C. Following careful aspiration of the MTT solution, 100 µL of DMSO were added to each well to dissolve the formazan crystals. The extent to which MTT was reduced to its formazan salt by hTERT-HCECs was assessed by SPECTROStar Nano^®^ absorbance microplate reader (BMG LABTECH, Ortenberg, Germany) measurements at 550 nm with prior shaking for 5 min. The negative control was a 0.9% *w/v* NaCl solution, and the positive control was an aqueous solution of 0.005% *w/v* BAK, both diluted 1:1 with supplemented cell culture medium. Although it is a component often included as a preservative in topical ophthalmic medications, BAK was selected as it is linked to inflammatory cascades in corneal and conjunctival cells [45, 46].

### In vivo studies

Normotensive albino male New Zealand rabbits (n = 11) were purchased from Granja San Bernardo (Tulebras, Spain); at the end of the study they had a mean weight of 3.24 ± 0.16 kg. The animals were housed in individual cages with free access to food and water and were maintained under 12 h light-dark cycles (lights on from 8:00 am to 8:00 pm) at a room temperature of 18 °C and at approximately 50% relative humidity in a controlled atmosphere. In vivo studies fulfilled the 3R principles and followed the Association for Research in Vision and Ophthalmology (ARVO) Statement for the Use of Animals in Ophthalmic Vision Research, European Council Directive (86/609/EEC) and the Spanish Regulation on Experimental Studies with Animals (RD 53/2013 of 1 February 2013, modified by RD 118/2021 of 23 February 2021). The Animal Experimentation Ethics Committee of the Complutense University of Madrid approved the study protocol under the code ES280790000086.

#### In vivo tolerance

B-HA-LIP and LAT-HA-LIP tolerance were assessed by topically administering 25 µL of each formulation in both animal eyes (n = 6 animals). 0.9% *w/v* NaCl and MF were used as controls following the same procedure (n = 6 animals). Evaluation was performed by a masked observer both before the instillations and 4 h later. Ocular signs were classified according to the Draize test (ISO 10993-10:2010). This test includes a scoring system that encompasses six distinct components of observable alteration, such as toxicity and inflammation, in the anterior segment of the eye; these elements include the density and area of corneal opacification, the severity of eventual iritis, conjunctival redness, oedema, and any discharge that may be produced (Table 2). Before beginning the study, the authors selected a total score equal to 10 as the cut-off for establishing good tolerance of the tested formulations [47]. Pictures of the rabbits’ eyes before and 4 h after each instillation were taken using a VX75 slit lamp (Luneau Technology, Chartres, France).

**Table 2:** Scoring system, according to the Draize test, employed to determine the in vivo tolerance of the liposomal formulations

CORNEA Score = A × B × 5 (range 0–80)	A. Opacity/Degree of density (most dense area taken for reading) - Scattered or diffuse area; details of iris clearly visible = 1 - Easily discernible translucent areas; details of iris slightly obscured = 2 - Opalescent areas; no details of iris visible, size of pupil barely discernible = 3 - Opaque; iris invisible = 4
	B. Area of cornea involved - One quarter (or less), but not zero = 1 - Greater than one quarter, less than one half = 2 - Greater than one half, less than three quarters = 3 - Greater than three quarters, up to whole area = 4
IRIS Score = A × 5 (range 0–10)	A. Values Folds above normal, congestion, swelling, and/or circumcorneal injection; iris still reacting to light (sluggish reaction is positive) = 1 No reaction to light, haemorrhage, and/or gross destruction = 2
CONJUNCTIVA Score = (A + B + C) × 2 (range 0–20)	A. Redness of palpebral conjunctiva - Vessels definitely injected above normal = 1 - More diffuse, deeper crimson red; individual vessels not easily discernible = 2 Diffuse beefy red = 3
	B. Chemosis - Any swelling above normal (includes nictitating membrane) = 1 - Obvious swelling with partial eversion of the lids = 2 - Swelling with lids about half closed = 3 - Swelling with lids about half closed to completely closed = 4
	C. Discharge - Any amount different from normal = 1 - Discharge with moistening of the lids and hairs adjacent to the lids = 2 - Discharge with moistening of the lids and considerable area around the eye = 3
Total score	= Sum of all scores obtained for the cornea, iris and conjunctiva

#### Hypotensive studies in rabbits

The efficacy of the proposed liposomal formulations (LAT-HA-LIP) in reducing IOP was assessed in a total of six rabbits (n = 12 eyes). The same protocol was followed for both MF and 0.9% *w/v* NaCl (n = 12 eyes each). The latter was used as a negative control to determine IOP fluctuations throughout the day due to circadian rhythms [48, 49]. MF was used as the benchmark for comparing changes in IOP over time as its effect is already described in clinical practice. Unloaded liposomes (B-HA-LIP) were also evaluated (n = 12 eyes). IOP measurements were taken with an iCare^®^ TonoVet TV01 rebound tonometer fitted with original iCare^®^ tonometer probes (V1089342/05/17, iCare Finland Oy, Vantaa, Finland). Each IOP measurement corresponded to the average of six correct single measurements and was taken by placing the tip of the probe at 4–8 mm and guaranteeing its contact with the centre of the cornea. To establish baseline IOP, two consecutive tonometric readings were performed at 30 min and immediately before instillation in each eye. After that, 25 µL of each tested formulation was gently instilled in both eyes of each rabbit. All the experiments began at the same time every day (9 am) to avoid the bias induced in IOP by circadian rhythms. Subsequently, IOP readings were logged every hour for a total of 11 h the first day, at 24, 28 and 32 h after instillation the following day, and then once per day until baseline IOP values were completely restored. Several parameters were set and calculated to analyse the results of these hypotensive studies: total IOP reduction percentage at each timepoint (ΔIOP), maximum IOP reduction percentage (IOP_max_), area under the IOP curve from the beginning of the study (t0) until the time of the measurement recorded prior to restoration of baseline IOP values (t’) (AUC_t0-t’_), time when each formulation started to be effective (t_onset_) and total IOP reduction time (t_effective_). AUC_t0-t’_ was estimated using the lineal trapezoidal rule. To determine whether use of the formulations enhanced bioavailability, a 95–105% interval was determined as per bioequivalence research guidelines [50, 51].

#### Latanoprost determination in rabbit tear fluid

LAT concentration in rabbit tear fluid (RTF) was evaluated for LAT-HA-LIP at three different timepoints: 10, 30 and 60 min (n = 4 eyes). A volume of 25 µL of LAT-HA-LIP was topically administered in the lower conjunctival sac. RTF was collected after each timepoint using sterile diagnostic Schirmer’s test strips (T213, Contacare Ophthalmics and Diagnostics, Gujarat, India), which were positioned in the inferior eyelid for a total of 60 s and then protected from light and stored in plastic tubes at − 80 °C until processing. The same procedure was performed to determine LAT concentration in RTF after topical MF administration (n = 4 eyes per timepoint). As a negative control, RTF was collected from untreated animals (n = 6) following the same protocol. Subsequently, LAT in RTF was determined after extraction of the test strips with acetonitrile-Milli-Q^®^ water in a 70:30% *v/v* ratio (500 µL in 1.5 mL plastic tubes). Afterwards, each sample was subjected to sonication (Sonorex Digiplus DL 510 H, Bandelin, Berlin, Germany) for 5 min and then centrifuged (5000 rpm, 5 min, 20 °C). Supernatants were analysed with the ultra-performance liquid chromatography (UPLC) line of a Waters^®^ Acquity Arc Bio UHPLC device. The chromatographic conditions were the same as described in 2.4. except for injection volume, which was set at 50 µL. Seven standard dilutions were prepared from a 5 mg mL^−1^ LAT stock solution in acetonitrile (0.18, 0.35, 0.70, 0.88, 1.16, 1.75, 3.50 µg mL^−1^) to obtain the regression line (y = 28,486.45·x + 2614.28, R^2^ = 0.9950). LOD and LOQ were respectively established at 0.09 µg mL^−1^ and 0.26 µg mL^−1^. To estimate LAT concentration, samples volume was set at 7.5 µL, considered equal to the total RTF [52].

### Statistical analysis

Each liposomal formulation was prepared in three separate batches and each batch was analysed in triplicate. Cytotoxicity data were collected from three separate experiments on three different days (biological replicates), and seven wells were tested for each sample (technical replicates) to ensure reproducibility. The results were expressed as a decrease in cell viability [%] relative to the negative control. Physicochemical, in vitro and hypotensive results were expressed as the mean ± standard deviation of the means (SD). In vivo tolerance determinations were reported as the mean ± standard error of the means (SEM). Unpaired *t*-tests were selected to determine statistical differences during physicochemical, in vivo tolerance, AUC_t0-t’_ comparisons and LAT determination in RTF assays. Two-way multivariate analysis of variance (ANOVA) using Šídák's multiple comparisons test was employed to compare results for the formulations tested in the cell viability studies. Differences between LAT-HA-LIP and MF in the hypotensive efficacy treatments were considered significant when the two-sided 95–105% confidence interval for the difference between the means of the selected parameters excluded zero [53]. GraphPad Prism^®^ software (version 9.5.0, GraphPad Software LLC) was used for the statistical determinations. A probability value lower than 0.05 (p-value < 0.05) was considered statistically significant.

## Results

### Physicochemical characterisation of the liposomes

**Fig. 2:**
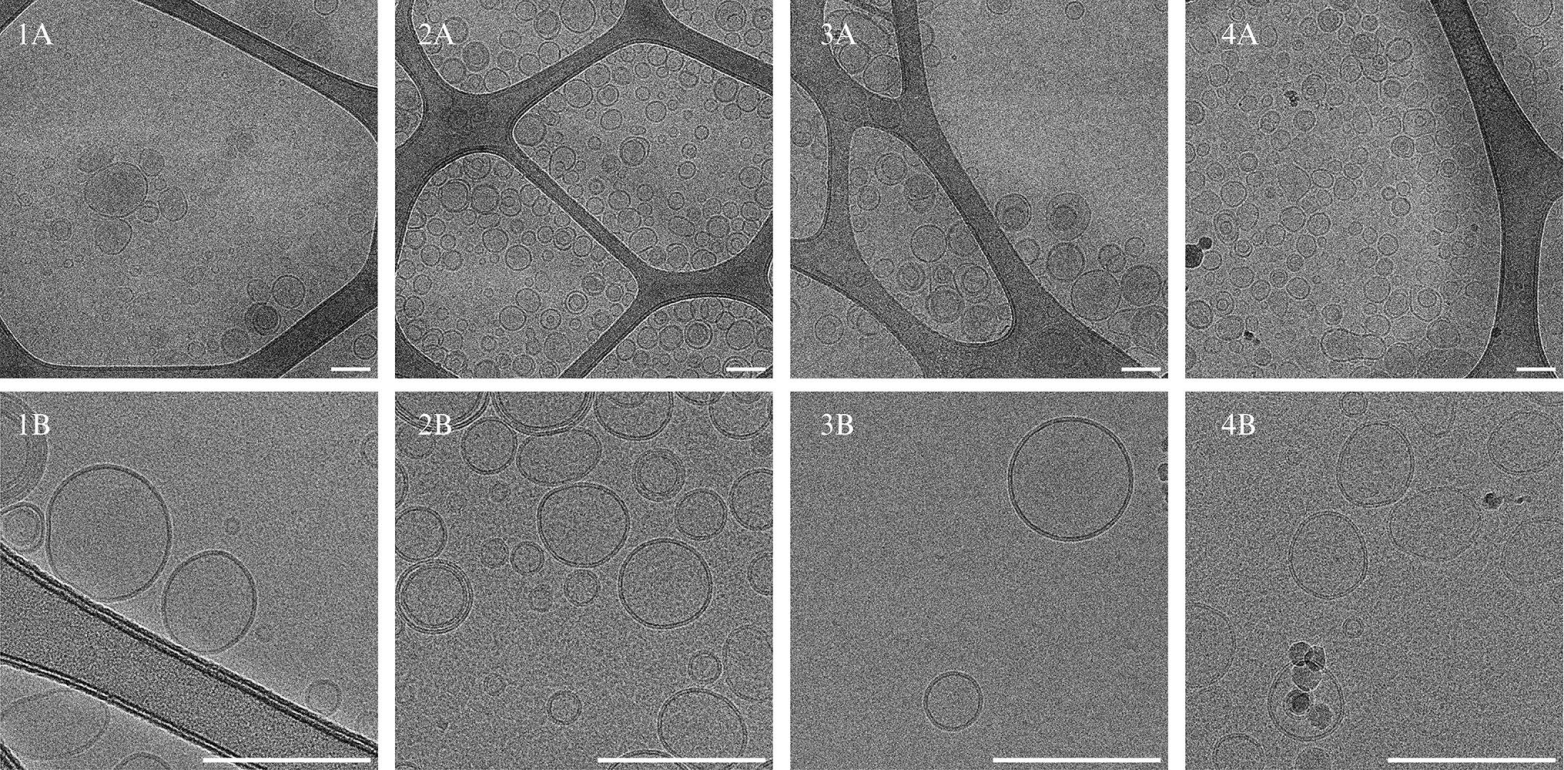
Representative images of B-LIP (**1**), B-HA-LIP (**2**), LAT-LIP (**3**) and LAT-HA-LIP (**4**) under cryo-EM at × 22,000 (A) and × 92,000 (B) magnifications. Scale bar: 200 nm

**Fig. 3:**
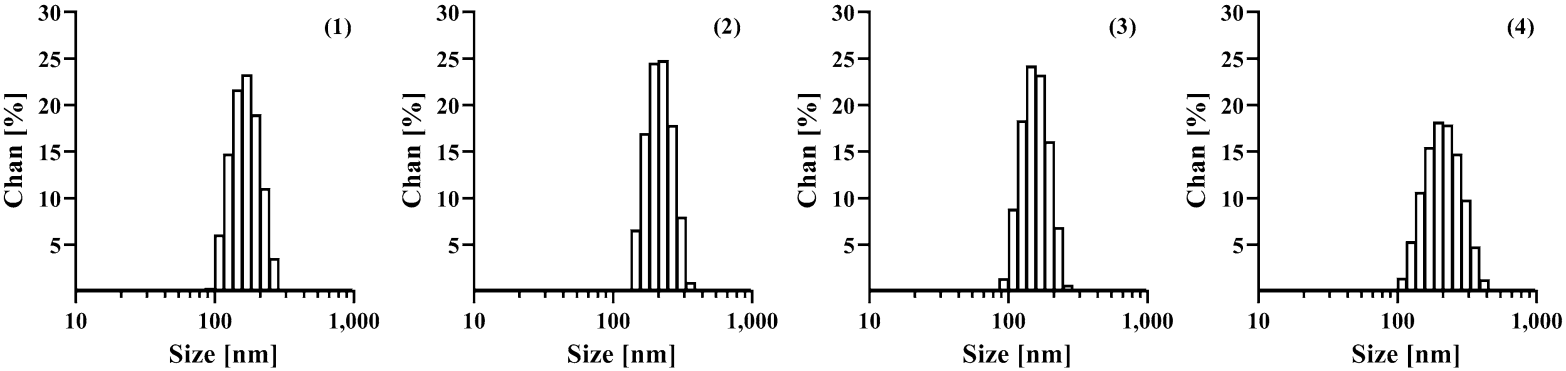
Vesicle size distributions for B-LIP (**1**), B-HA-LIP (**2**), LAT-LIP (**3**) and LAT-HA-LIP (**4**)

Under cryo-EM visualisation, all the developed liposomes presented mostly spherical-shape unilamellar vesicles and homogeneous sizes that correlated with those obtained by the dynamic light-scattering technique (Fig. 2). The four liposomal formulations shared a unimodal distribution and exhibited a narrow unimodal particle size distribution (Fig. 3). The average sizes for the four formulations ranged between 150 and 200 nm. LAT inclusion did not affect vesicle size (p > 0.05) with or without 0.2% *w/v* HA in the vehicle. However, mean particle size increased in mucoadhesive formulations containing 0.2% *w/v* HA (B-HA-LIP and LAT-HA-LIP). The zeta potential of formulations without 0.2% *w/v* HA was found to be neutral, as it fell between -10 mV and 10 mV. Nonetheless, the zeta potentials of B-HA-LIP and LAT-HA-LIP were moderately more negative than those of formulations without the mucoadhesive polymer. That is also reflected in the conductivity results; for instance, B-HA-LIP showed slightly higher conductivity (0.093 mS cm^−1^) than B-LIP (0.070 mS cm^−1^). The four formulations shared similar pH values (7.63–7.67) compatible with ocular surface pH. Surface tension was lower for formulations in which the mucoadhesive polymer was not included than it was in the ones with 0.2% *w/v* HA. Rheological studies revealed that B-LIP and LAT-LIP exhibited similar values to water and human tears (close to 1.00 mPa s^−1^), while viscosity was more than 2.5 times higher for the formulations in which 0.2% *w/v* HA was dispersed in the vehicle. The results for the parameters evaluated are listed in Table 3.

**Table 3:** Characterisation of B-LIP, B-HA-LIP, LAT-LIP and LAT-HA-LIP by size, polydispersity index (PDI), zeta potential, pH, surface tension, osmolarity and viscosity

Formulation	Size [nm]	PDI	Zeta potential [mV]	pH	Surface tension [mN m^−1^]	Osmolarity [mOsm L^−1^]	Viscosity [mPa s^−1^]
B-LIP	157.97 ± 9.35	0.17	-5.73 ± 0.57	7.65 ± 0.01	21.30 ± 0.36	278.33 ± 2.05	1.09 ± 0.05
B-HA-LIP	209.52 ± 19.63	0.09	-14.51 ± 0.61	7.63 ± 0.01	47.60 ± 2.17	283.00 ± 2.16	2.56 ± 0.06
LAT-LIP	151.00 ± 10.88	0.09	-7.07 ± 0.87	7.67 ± 0.01	28.50 ± 0.62	280.33 ± 0.47	1.10 ± 0.01
LAT-HA-LIP	195.14 ± 14.34	0.15	-13.96 ± 0.78	7.63 ± 0.01	44.07 ± 2.70	284.00 ± 1.41	2.69 ± 0.15

### Quantitation of the latanoprost in the liposomal formulation

LAT was quantified in terms of LAT_tot_ and EE. LAT_tot_ was determined being equal to 52.26 ± 2.05 µg mL^−1^ (104.52 ± 4.10%), the same nominal concentration reported in MF. Since no chromatographic peak was detected in the ultrafiltrate, LAT_free_ was below the LOD, which means 0.54% over LAT_tot_. Therefore, EE was assured to be greater than or equal to 99.46%. This result indicates that all the LAT included was incorporated inside the synthetic phospholipids’ lamellae.

### In vitro studies in human corneal and conjunctival cells

**Fig. 4:**
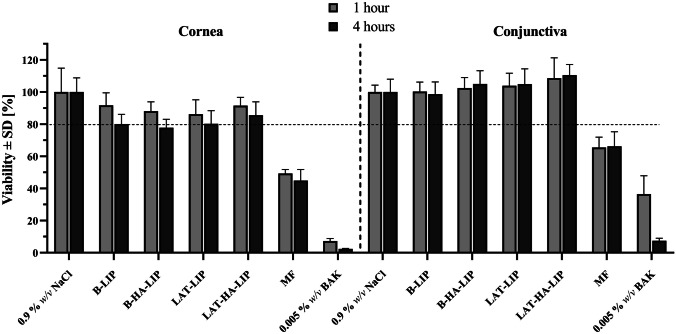
In vitro tolerance of the liposomal formulations developed in corneal (hTERT-HCECs) and conjunctival (IM-HConEpiC) cells, compared with MF, a negative control (0.9% w/v NaCl) and a positive control (0.005% w/v BAK), at 1 h and 4 h of cell exposure to the formulations

Regarding the viability studies in the corneal cell line (hTERT-HCECs), B-LIP, B-HA-LIP, LAT-LIP and LAT-HA-LIP all presented values higher than 80% at both exposure times (1 h and 4 h) with no statistical difference found between them (p > 0.05), indicating optimal tolerance (Fig. 4). The liposomal formulations also presented relevantly higher tolerance results than MF. Similar results were obtained when formulation tolerance was assayed in a conjunctival cell line (IM-HConEpiC). B-LIP, B-HA-LIP, LAT-LIP and LAT-HA-LIP all showed cell viabilities higher than 80%, a result that MF did not achieve. The addition of 0.2% *w/v* HA seemed to produce a slight improvement in cell viability, although the variance from the liposomal formulation without the mucoadhesive polymer was not statistically significant (p > 0.05).

### In vivo studies

#### In vivo tolerance

**Fig. 5:**
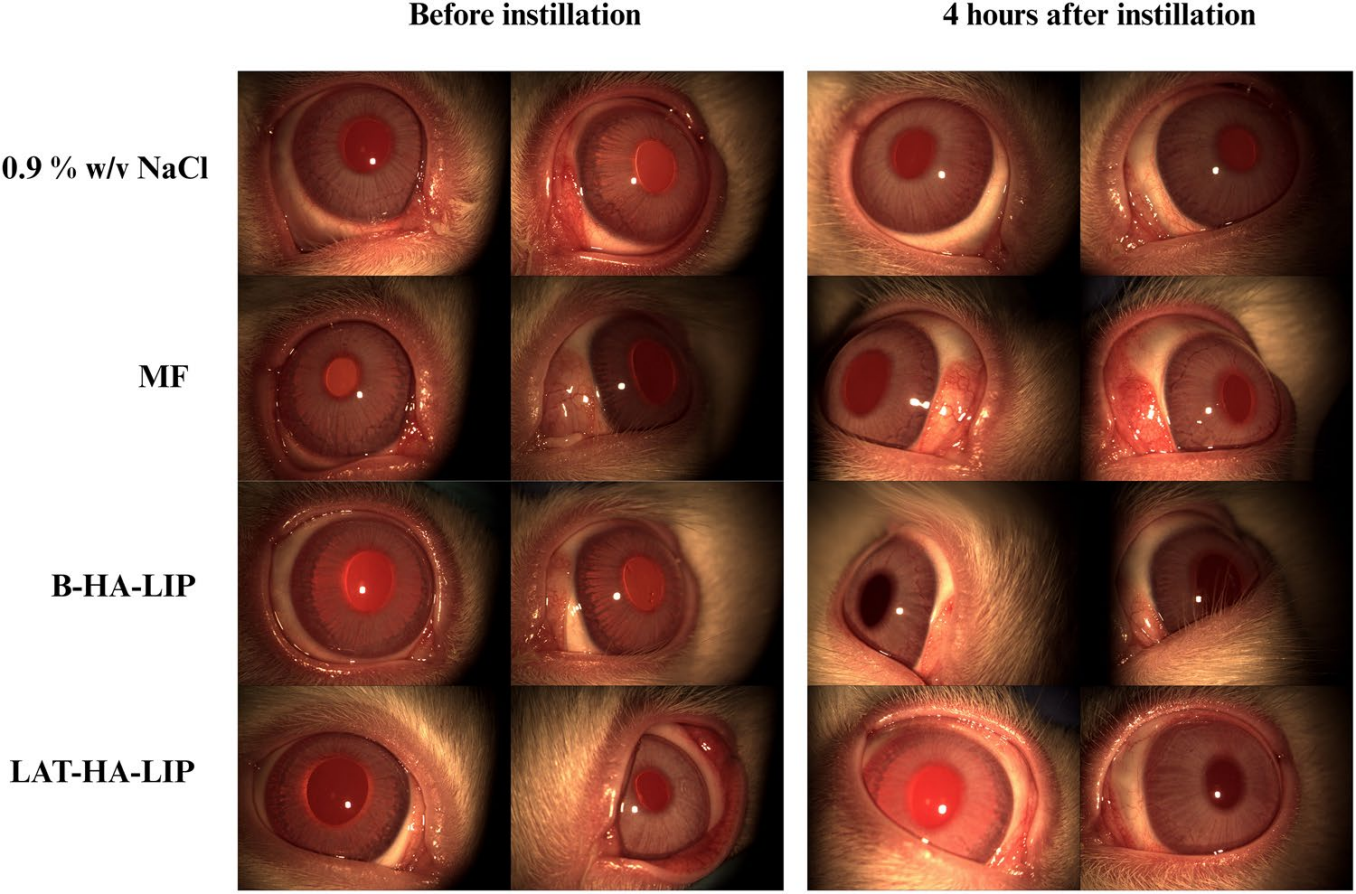
Representative slit lamp images of the formulations evaluated for rabbit eye tolerance

The in vivo tolerance study in albino male New Zealand rabbits (n = 12 eyes per formulation) found no signs of ocular surface damage or pathological changes after administration of either B-HA-LIP or LAT-HA-LIP (Fig. 5). Each animal was evaluated before instillation by summing all the parameters as stated in the Draize test (1.00 ± 0.30 for 0.9% *w/v* NaCl, 0.67 ± 0.28 for MF, 1.17 ± 0.39 for B-HA-LIP and 1.50 ± 0.36 for LAT-HA-LIP). No score was recorded in the course of the experiment in the ‘corneal opacity’, ‘iritis’ and ‘chemosis’ sections as none of these signs were macroscopically observed. All the formulations administered produced a total score significantly lower than the cut-off established at 4 h after each instillation (0.67 ± 0.28 for 0.9% *w/v* NaCl, 3.50 ± 0.78 for MF, 0.33 ± 0.22 for B-HA-LIP, 2.17 ± 0.39 for LAT-HA-LIP). No score greater than 1 was recorded for ‘discharge’ in all the formulations assayed. However, scores equal to 2 and 3, indicating a diffuse redness of palpebral conjunctiva, were observed at 4 h after MF administration. Although the totals were slightly lower, according to the Draize test LAT-HA-LIP was tolerated as well as MF (p > 0.05).

#### Hypotensive studies in rabbits

**Fig. 6:**
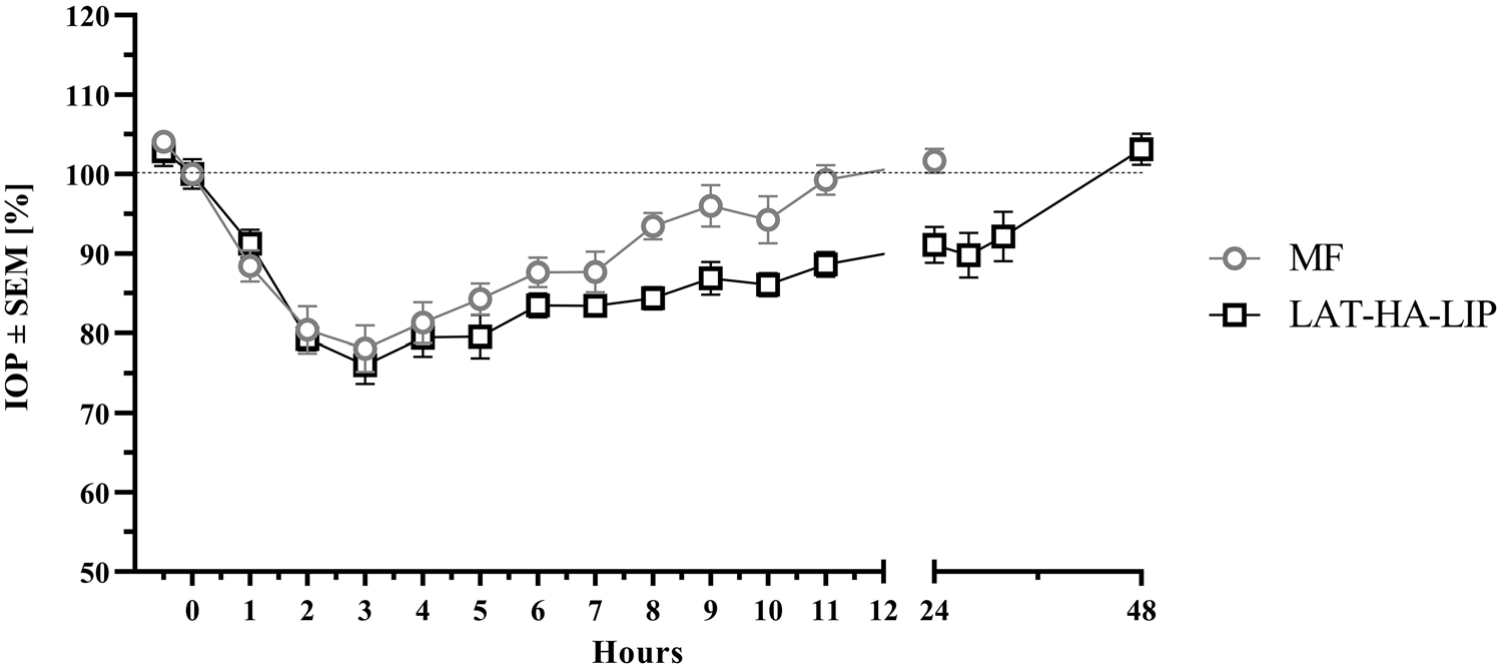
Comparison of hypotensive efficacy between LAT-HA-LIP and MF. IOP values are expressed in decreasing percentage versus baseline, set at 100%

The hypotensive effect of the developed liposomes dispersed in the mucoadhesive polymer (LAT-HA-LIP) was measured and compared to MF (Table 4). IOP values of rabbits in which B-HA-LIP was instilled over the test period were not relevantly different to those in which 0.9% *w/v* NaCl was instilled. LAT-HA-LIP exhibited noticeably longer hypotensive activity than MF (Fig. 6). The hypotensive effect of MF persisted for 24 h, whereas the hypotensive activity of LAT-HA-LIP lasted until 48 h after instillation. IOP results are shown in Table 4. Although t_onset_ was the same and observed at 1 h for both formulations (p > 0.05), t_effective_ was 1 day longer for LAT-HA-LIP than for MF. Furthermore, IOP_max_ was recorded 3 h after instillation for both formulations; no statistical difference was observed between LAT-HA-LIP and MF (p > 0.05) as reflected by the 95% confidence interval of the IOP_max_ (-4.41% < LAT-HA-LIP – MF < 8.56%) (Fig. 2S). While IOP returned to normal values 24 h after MF instillation, at the same timepoint LAT-HA-LIP presented a significantly different hypotensive effect (p = 0.0024) versus the 100% baseline value. The 24-h longer hypotensive effect also resulted in a significantly higher LAT-HA-LIP area under the IOP curve (AUC_t0-t’_) versus that of MF (p = 0.0004) (Fig. 1S). The efficacy of the formulations, as calculated by the 95% confidence interval of the AUC_t0-t'_ (177.58%·h < LAT-HA-LIP – MF < 329.07%·h) was significantly higher for LAT-HA-LIP than MF (Fig. 2S).

**Table 4:** Parameters evaluated in hypotensive studies in rabbits

Formulation	ΔIOP (t_onset_) [%]	ΔIOP (24 h) [%]	IOP_max_ [%]	AUC_t0-t’_ [%·h]
MF	11.57 ± 6.82	-1.68 ± 5.27	21.94 ± 10.14	128.80 ± 43.85
LAT-HA-LIP	8.75 ± 6.17	8.92 ± 7.89	24.02 ± 8.26	382.13 ± 146.36

#### Latanoprost determination in rabbit tear fluid

**Fig. 7:**
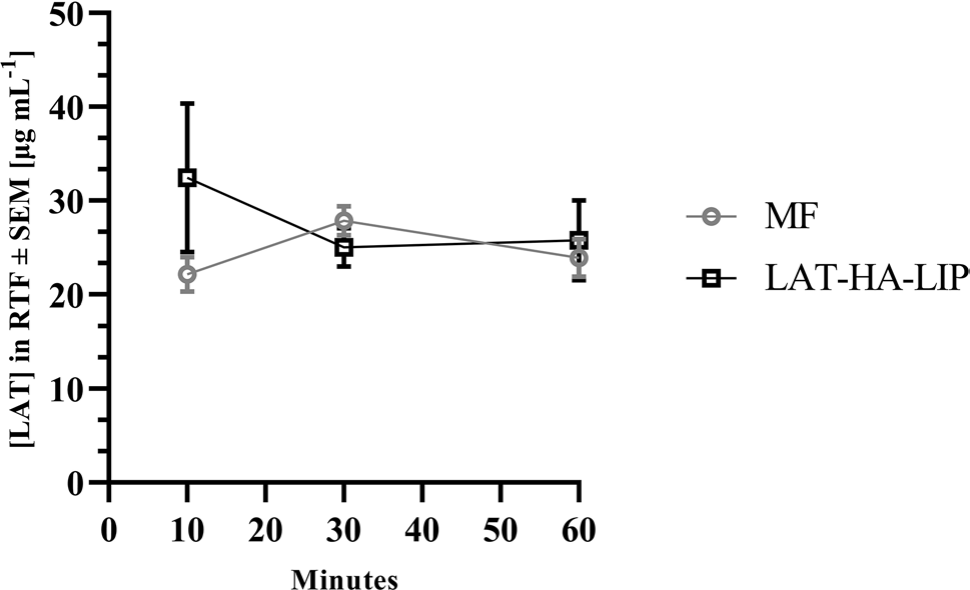
LAT concentration in RTF at 10, 30 and 60 min after instillation of LAT-HA-LIP and MF (n = 4)

LAT concentration in RTF was quantified at 10, 30 and 60 min after separate administration of LAT-HA-LIP and MF, respectively (Fig. 7). LAT was properly extracted from Schirmer’s test strips and determined by the regression equation established by UPLC analyses, as described in the methodology (Fig. 3S). After analysing RTF collected from untreated animals, no chromatographic peaks were observed at the same LAT retention time, thus confirming the selectivity of LAT in RTF-treated rabbits. No statistical difference (p > 0.05) was observed following LAT quantification in RTF at 10 min (32.45 ± 11.21 µg mL^−1^ for LAT-HA-LIP and 22.15 ± 3.16 µg mL^−1^ for MF), 30 min (25.00 ± 4.12 µg mL^−1^ for LAT-HA-LIP and 27.86 ± 2.71 µg mL^−1^ for MF) and 1 h (25.75 ± 7.37 µg mL^−1^ for LAT-HA-LIP and 23.89 ± 3.97 µg mL^−1^ for MF) after topical ophthalmic formulation administration.

## Discussion

Liposomes offer promising prospects for delivering therapeutic agents to the ocular surface. These biocompatible and biodegradable drug delivery systems have been widely researched as possible antiviral, antibacterial, antifungal and immunological therapies [54–57]. In ophthalmic administration, liposomes have been used to reduce the toxicity and adverse effects of therapeutic molecules while increasing their efficacy and, consequently, enhancing patient adherence to treatment [19]. In the case of glaucoma, several efforts have already been made to include hypotensive agents – acetazolamide, methazolamide, brinzolamide and timolol maleate, among others – using phosphatidylcholine derivatives of natural origin (soy or egg) [39, 58–60]. Liposomal formulations loaded with prostaglandin analogues have been developed to be topically applied or injected at subconjunctival level [61–63]. Several attempts have been made to date to include prostaglandin analogues, such as travoprost or LAT, in nanovesicles composed of synthetic phospholipids [63, 64].

This paper focuses on using several technology-based strategies to increase LAT efficacy based on liposomes’ development as drug delivery systems in glaucoma therapy. These approaches aim not only to increase ocular bioavailability following topical ophthalmic administration but also to protect the ocular surface against potential damage during chronic topical glaucoma treatment with prostaglandin analogues, a therapy extensively described in the literature as correlated with DED [65]. MF, approved in 2013, was employed in this paper for comparing in vitro and in vivo tolerance and the therapeutic effects. This ophthalmic solution, devoid of BAK, features a 5% *w/v* concentration of the non-ionic surfactant macrogolglycerol hydroxystearate 40, along with Carbomer 974 P and Macrogol 4000, sorbitol, disodium edetate and sodium hydroxide for pH adjustment [66]. Due to their lipophilic nature, additional excipients must be incorporated into the eye drop solutions to enhance the solubility and stability of prostaglandin analogues, such as LAT. Concurrently, these excipients may also inadvertently cause adverse effects on the ocular surface when administered repeatedly [67, 68]. Based on our understanding, this study constitutes the first attempt to obtain the long-lasting hypotensive effect of LAT while simultaneously protecting the ocular surface against the damage associated with its chronic use. To our knowledge, there is no liposome-based formulation on the market for glaucoma treatment.

To this end, LAT was included in nanovesicles mainly composed of neutral synthetic phospholipids (namely DOPC and DMPC) along with cholesterol to confer rigidity [69, 70]. As cited previously, regardless of the extensive use of soy or egg phosphatidylcholine as a main component of liposomal formulations, employing synthetic phospholipids has the advantage of avoiding reproducibility issues due to their purity and homogeneous saturated (DMPC) or unsaturated (DOPC) fatty acid compositions [71, 72]. Phase transition temperature (T_c_), specifically the temperature at which a phospholipid shifts from an ordered and rigid ‘gel’ state to a disordered and fluid ‘sol’ state, was the parameter considered for combining DOPC (T_c_ ≈ -17 °C) and DMPC (T_c_ ≈ 24 °C) [73, 74]. DMPC was added to the synthetic phospholipid mixture to bring it closer to the ‘gel’ state, thus increasing vesicle stability once administered at ocular surface temperature (≈ 32–34 °C) [44, 64]. In addition, α-tocopherol acetate was included in the liposome preparation process to prevent lipid peroxidation [75]. As cited previously, use of synthetic phospholipids has been widely described. In fact, González-Cela et al. reported promising results in extending IOP decrease up to 48 h after single administration of travoprost-loaded liposomes, achieving an area under the hypotensive curve 1.7 times greater than with a commercial travoprost medication [64]. Fahmy et al. subconjunctivally injected LAT-loaded liposomes consisting of 1,2-dipalmitoyl-sn-glycero-3-phosphocholine (DPPC) in albino male New Zealand rabbits in which glaucoma had been induced. Their drug delivery system provided in vivo sustained LAT release and showed maximum IOP-reduction effect up to 84 h when compared with other tested formulations [63].

As demonstrated by HPLC-UV analyses, the entire LAT initially included in manufacture (LAT_tot_) was detected and quantified in the formulation and fully incorporated inside the lipid bilayer, as the EE obtained was close to 100%. LAT_tot_ concentration was similar to the nominal concentration present in MF (0.005% *w/v* LAT), thus meaning it was possible to compare the efficacy of the two formulations for the same administered dose.

The liposomal vesicles proposed in this paper had suitable sizes and zeta potential, critical criteria for creating stable colloidal dispersions, avoiding ocular irritation and lengthening their binding with the conjunctiva and cornea. A vesicle size of around 200 nm or smaller, like the formulations developed in this study, is desirable as it allows liposomes to permeate the corneal epithelium layers to decrease immunogenicity, to circumvent mononuclear phagocytic system uptake and, finally, to avoid blurred vision after instillation [76, 77].

Given tears’ physicochemical characteristics, topical ophthalmic formulations must meet several specific criteria. Key parameters among these are pH, surface tension, osmolarity and viscosity, which play a crucial role in developing treatments for compromised ocular surfaces. All the developed formulations presented optimal pH for ocular surface comfort and safety (which, physiologically, is approximately 7.4). This parameter was maintained by use of a borate buffer which, as reported in the literature, also helps preserve the formulation against contaminants [78]. In the aqueous dispersion prepared, TREH was included to achieve the optimal isotonic osmolarity (≈ 290 mOsm L^−1^). Moreover, it was already included in marketed available artificial tears and described as behaving like an osmoprotectant for its ability in enhancing autophagic flux when in combination with HA [79–81]. Surface tension was similar to the values observed in tears (43.60 ± 2.70 mN m^−1^) capable of ensuring good liposomal formulation extension on the ocular surface [82, 83]. In addition, isotonic formulations were measured in all cases to ensure the absence of discomfort after instillation. Regarding rheological studies, formulations including 0.2% *w/v* HA in the vehicle showed almost three-times higher viscosities than the ones prepared without the polymer. In all formulations, viscosity values were compatible with those of natural tears (0.3–8.3 mPa s^−1^), thus avoiding blurred vision and allowing uniform mixing with the tear film [19]. These afore-mentioned physicochemical characteristics were found to resemble the parameters exhibited by the benchmark. As previously described, MF had a pH value (6.84 ± 0.03) close to the one of the ocular surface, showed to be isotonic (275.8 ± 0.7 mOsm L^−1^) and with optimal surface tension for ensuring a stable tear film and tear film break-up time (42.44 ± 0.75 mN m^−1^) [84].

Cryo-EM proved to be the technique of choice to visualise this kind of drug delivery system without inducing size modifications [85, 86]. Cryo-EM visualisation revealed that all the liposomal formulations conserved spherical vesicle-like morphologies and that the addition of HA did not modify their structure. Our results were consistent with findings previously reported by our research group [38].

The addition of 0.2% *w/v* HA promoted a slight increase in vesicle size. This result could be explained by the physical coating of the anionic biopolymer, a phenomenon consistent with other the findings of previously published studies [87–90]. The physical adhesion of the HA chains around the liposomes could also explain the reduction in the zeta potential observed, as other authors have also pointed out [91]. These conclusions could confirm our hypothesis regarding the mucoadhesive nature of B-HA-LIP and LAT-HA-LIP formulations, the zeta potential of which was slightly lower than that measured in formulations in which the polysaccharide was not included.

The osmoprotective substances, such as BET and LEU, were included to protect the ocular surface from the hyperosmolar stress occurring as a consequence of potential preocular tear film destabilisation caused by chronic antiglaucoma treatment. As previously described, the use of BET has been shown to stabilise corneal epithelial cell volume under hyperosmotic stress and to limit cell apoptosis. In addition, when topically administered it proved effective in reducing DED progression [31, 92]. Similarly, LEU exhibited anti-inflammatory effects [33]. The above-mentioned combination of osmoprotective substances (0.40% *w/v* BET and 0.90% *w/v* LEU), jointly included with 0.2% *w/v* HA, was dissolved in a borate buffer vehicle to tailor the formulation for ocular instillation. A study recently published by our research group showed that the synergistic action of the three compounds protected hTERT-HCECs after chronic exposure to hyperosmotic solutions (between 450 and 500 mOsm L^−1^) [34].

Mucoadhesive properties of hyaluronic acid have not been demonstrated in the present article, however their ability in interacting with mucins and in increasing the retention time of topical ophthalmic formulations onto the ocular surface have been previously explored [93, 94]. Moreover, in a publication of our research group, the interfacial interaction of hyaluronic acid 0.2% w/v with isolated corneal transmembrane mucins was evaluated using a novel in vitro surface biosensor technique. In this study, the mechanism of mucoadhesion for hyaluronic acid was suggested to be related with physical chain interpenetration with the named mucins on the ocular surface [95]. In this present work, the mucoadhesive polymer HA was included to increase formulation retention time on the ocular surface [19, 96]. In fact, incorporation of viscous HA solution in medicated eye drops based on liposomes has been previously implemented, yielding promising results. For instance, Quinteros et al. observed that when the hypotensive melatonin analogue 5-methoxycarbonylamino-N-acetyltryptamine was loaded in soy phosphatidylcholine liposomes and dispersed in 0.2% *w/v* HA it induced remarkably higher IOP reduction (39.13 ± 2.21%) when topically administered in albino male New Zealand rabbits than other liposomes formulated with or without other mucoadhesive polymers like carboxymethylcellulose (36.72 ± 2.77%) and a mixture of poloxamer 407 and 188 (29.12 ± 2.11%); additionally, the formulation exhibited excellent in vivo tolerance, with the hypotensive effect lasting more than 8 h [97].

In this study, the mucoadhesive properties of HA probably facilitate LAT accumulation in the corneal and conjunctival epithelium, a crucial factor for effective transportation to the anterior segment of the eye; this accumulation should ensure prolonged LAT delivery to the targeted site of action [28]. This fact was observed in LAT concentration in RTF, determined over 1 h following LAT-HA-LIP and MF instillation. Although a mucoadhesive compound was included in MF formulation (e.g. Carbomer^®^ 974 P) and their difference was not statistically significant (p = 0.2041), LAT concentrations in RTF at 10 min after administration were appreciably higher for LAT-HA-LIP than for MF (32.45 ± 11.21 µg mL^−1^ for LAT-HA-LIP and 22.15 ± 3.16 µg mL^−1^ for MF). According to previous studies, the presence of HA in the vehicle counteracts rapid elimination from the ocular surface, slows the tear turnover rate and increases the retention time of the hypotensive liposomal formulation [27, 28]. Besides, since no active molecule (LAT free acid) was detected in the RTF samples, it can be hypothesised that over the period evaluated there was no esterases activity in the RTF, or if there was it was not enough to achieve LOD concentration.

Tolerance studies were performed on corneal and conjunctival epithelial cell cultures under simulated conditions of acute (1 h exposure) and chronic (4 h exposure) treatment previously established and validated by our research group [39]. In vitro tolerance of all four liposomal formulations was considerably higher at the two exposure times evaluated then it was for MF. These data support the translational manufacture of these liposomal formulations as a possible glaucoma treatment since they could be capable of maintaining the integrity of the ocular surface. Previous studies reported that poor tolerance of preservative-free MF could be explained by the presence of the solubilising agent and non-ionic surfactant macrogolglycerol hydroxystearate 40 in the composition of the ophthalmic solution, in addition to the inherent long-term side effects of LAT [68, 98, 99]. However, the LAT-loaded synthetic phosphatidylcholine liposomes combined with HA and osmoprotectants developed in this paper produced cell viability values higher than 80%, thereby demonstrating optimal in vitro tolerance. Addition of HA to ophthalmic formulations has been shown to increase the cell viability of topical formulations. Vicario de la Torre et al. proved that unloaded liposomes composed of soy phosphatidylcholine dispersed in 0.2% *w/v* HA yielded viability values higher than 95% in immortalised human corneal-limbal epithelial cells and human conjunctival cells after short and long exposure times versus formulations in which the mucoadhesive polymer was not included [37]. Similarly, Landucci et al. revealed that 0.1% *w/v* HA-coated liposomes composed of egg phosphatidylcholine were better tolerated (p < 0.01) than free thymoquinone at the same concentration on human corneal and conjunctival epithelial cells after 1 h of exposure time. Moreover, the same authors proved that a fluorescent formulation in which the mucoadhesive polymer was included improved in vitro cell uptake in the same cells of the hydrophobic drugs. Moreover, this was more pronounced than the permeation effect of liposomes without 0.1% *w/v* HA [100]. In this study, the addition of HA produced a slight increase in cell viability, albeit not one that was statistically significant compared to other treatments. This could be explained by the presence of the osmoprotectants in the formulations.

The in vivo tolerance of both B-HA-LIP and LAT-HA-LIP was assessed 4 h after instillation in albino male New Zealand rabbits as per the Draize test parameters. Promising results were revealed as no significant signs of discomfort or ocular surface damage were observed, thereby indicating the short-term safety of the developed preservative-free liposomal formulations. Minor signs of conjunctival hyperaemia were noticed when LAT-HA-LIP and MF were instilled, which could be linked to the presence of LAT itself [101, 102]. It should be underlined that none of the formulations assayed throughout the in vivo tolerance test, including MF, contained any preservatives, endorsing the in vitro tolerance results. Long-term studies would be required to assess significant changes in tolerance between the developed formulations and MF in chronic applications.

In vivo efficacy studies compared the hypotensive effect of LAT-HA-LIP to that of MF. Maximum IOP reduction (IOP_max_) was recorded 3 h after both LAT-HA-LIP and MF instillation, findings consistent with previous observations of LAT ocular pharmacokinetics [12, 103]. The hypotensive effect of the latter was maintained for 48 h, significantly outperforming the 24-h effect of the commercial formulation. Focusing on area under the IOP curve data (AUC_t0-t’_), LAT-HA-LIP exhibited 2.97 times greater relative ocular bioavailability than the reference formulation MF (p = 0.0006), thus meaning that LAT encapsulation inside synthetic phospholipid liposomes as presented here had a greater and longer hypotensive effect. This outcome has been hypothesised to occur due to the two technological approaches evaluated in this paper: i) the development of new LAT-loaded liposomal drug-delivery systems and ii) the presence of the mucoadhesive polymer. The findings of this paper concur with those of Fathalla et al., who incorporated LAT-loaded liposomes composed of soy phosphatidylcholine into three different gels (hydroxypropyl methylcellulose, Pluronic^®^ F-127 and Carbopol^®^ 934). While albino male New Zealand rabbits receiving the commercial formulation Xalatan^®^ returned to their IOP baseline values after 24 h, animals treated with the liposomal formulation dispersed in Pluronic^®^ F-127 showed a prolonged hypotensive effect over 72 h. This effect was attributed to the drug having an increased ocular residence time [61]. This same hypothesis was previously explored by our research group. Although the design of the formulation was different (travoprost was the hypotensive active, taurine and ribitol the osmoprotective agents and ubiquinol the antioxidant), synthetic phosphatidylcholine liposomes were dispersed in a hydroxypropyl methylcellulose (HPMC) 0.2% w/v viscous solution to take advantage of its mucoadhesive properties. In that work, the liposomal formulation including the mucoadhesive polymer HPMC, revealed a more gradual and prolonged effect compared to the benchmark employed [64].

To our knowledge, there is currently no commercially available prostaglandin analogue eye drop formulation that can guarantee the long-lasting hypotensive effect from a single application and the ocular surface tolerability offered by the proposed LAT-HA-LIP formulation. All these factors could improve patient adherence to chronic glaucoma therapy. Moreover, the ocular surface would be protected against components, such as the active substance, which could potentially trigger DED.

## Conclusions

This study focuses on enhancing latanoprost bioavailability and its hypotensive effect and on preventing the detrimental effects associated with its chronic administration. To this end, several technological strategies were employed: i) inclusion of latanoprost in synthetic phosphatidylcholine liposomes, which act as drug delivery systems, ii) addition of hyaluronic acid for its mucoadhesive properties and to increase residence time on the ocular surface, and iii) design of a vehicle capable of protecting ocular surface cells from the potential damage caused by chronic antiglaucoma treatments. The four liposomal formulations developed showed outstanding tolerance in corneal and conjunctival epithelial cells that was significantly superior to that of the commercially available latanoprost formulation used as the benchmark throughout this paper. One single eye drop of latanoprost-loaded liposomal formulation including hyaluronic acid (0.2% *w/v*) and the osmoprotectants betaine (0.40% *w/v*) and leucine (0.90% *w/v*) achieved a hypotensive effect that lasted 24 h longer and offered almost three times higher relative ocular bioavailability than the commercial medication. These results proved that the technological approaches described in this article increased the effect of the latanoprost and that the well-tolerated liposomal formulation developed constitutes a possible compliance-friendly candidate for ocular hypertensive treatment.

## Supplementary Information

Below is the link to the electronic supplementary material.

Supplementary file1 (DOCX 218 KB)

## Data Availability

Datasets and graphs are available from the corresponding authors upon request.
