# Supplementary material for: Enhancing the hypotensive effect of latanoprost by combining synthetic phosphatidylcholine liposomes with hyaluronic acid and osmoprotective agents
Source: Drug Deliv Transl Res. 2024 Apr 11;14(10):2804–22. doi: 10.1007/s13346-024-01584-z (PMC11385046; doi:10.1007/s13346-024-01584-z)
Supplement: Supplementary file 1 — Supplementary file1 (DOCX 218 KB) [file 13346_2024_1584_MOESM1_ESM.docx]

**Enhancing the Hypotensive Effect of Latanoprost by Combining Synthetic Phosphatidylcholine Liposomes with Hyaluronic Acid and Osmoprotective Agents**

Marco Brugnera^1,2,3^, Marta Vicario de la Torre^1,2,3^, Miriam Ana González-Cela Casamayor^1,2^, José Javier López Cano^1,2^, Irene Bravo Osuna^1,2,3^, Fernando Huete Toral^4^, María Luisa González Rubio^4^, Gonzalo Carracedo^4^, Irene Teresa Molina Martínez^1,2,3^, Vanessa Andrés Guerrero^1,2,3*^ and Rocío Herrero Vanrell^1,2,3*^

^1^ Innovation, Therapy and Pharmaceutical Development in Ophthalmology (InnOftal) Research Group, Complutense University of Madrid (UCM), Madrid, Spain (rociohv@ucm.es)

^2^ Department of Pharmaceutics and Food Technology, Faculty of Pharmacy, UCM; IdISSC, Madrid, Spain

^3^ University Institute of Industrial Pharmacy (IUFI), Faculty of Pharmacy, UCM, Madrid, Spain

^4^ Ocupharm Research Group, Department of Optometry and Vision, Faculty of Optics and Optometry, UCM, Madrid, Spain

** corresponding authors*

# Supplementary information


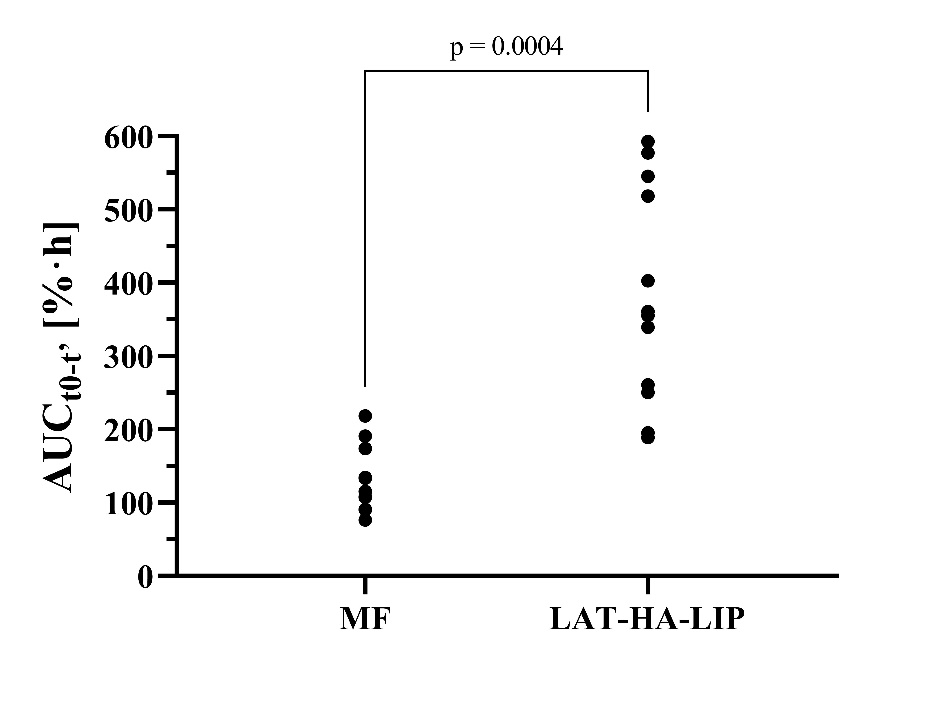


*Figure 1S. Comparison of AUC_t0-t’_ between MF and LAT-HA-LIP.*


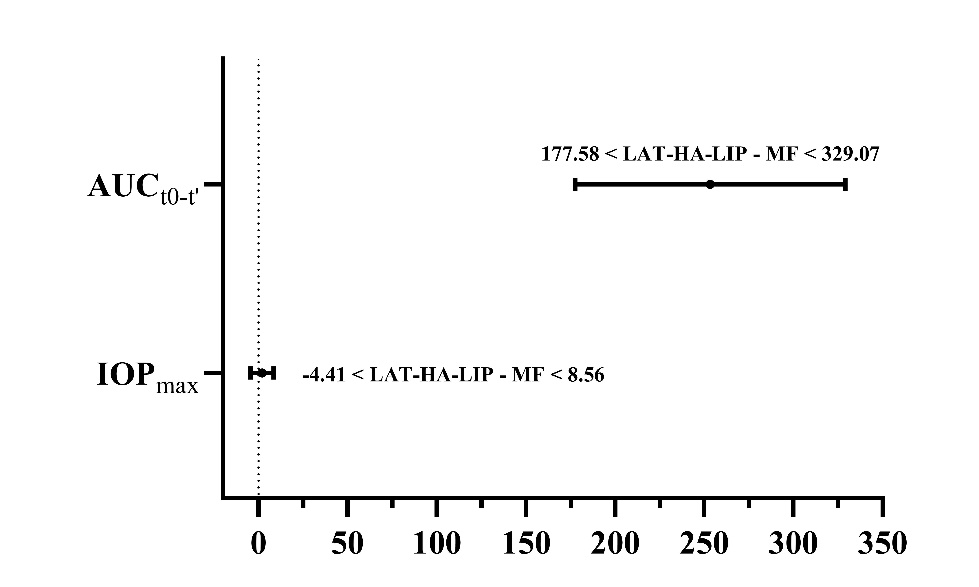


*Figure 2S. Relationship between the two mean difference tests and confidence intervals for the difference between the LAT-HA-LIP and MF means regarding the IOP_max_ and AUC_t0-t’_ parameters. Values to the right of zero indicate a better response to LAT-HA-LIP than to MF.*


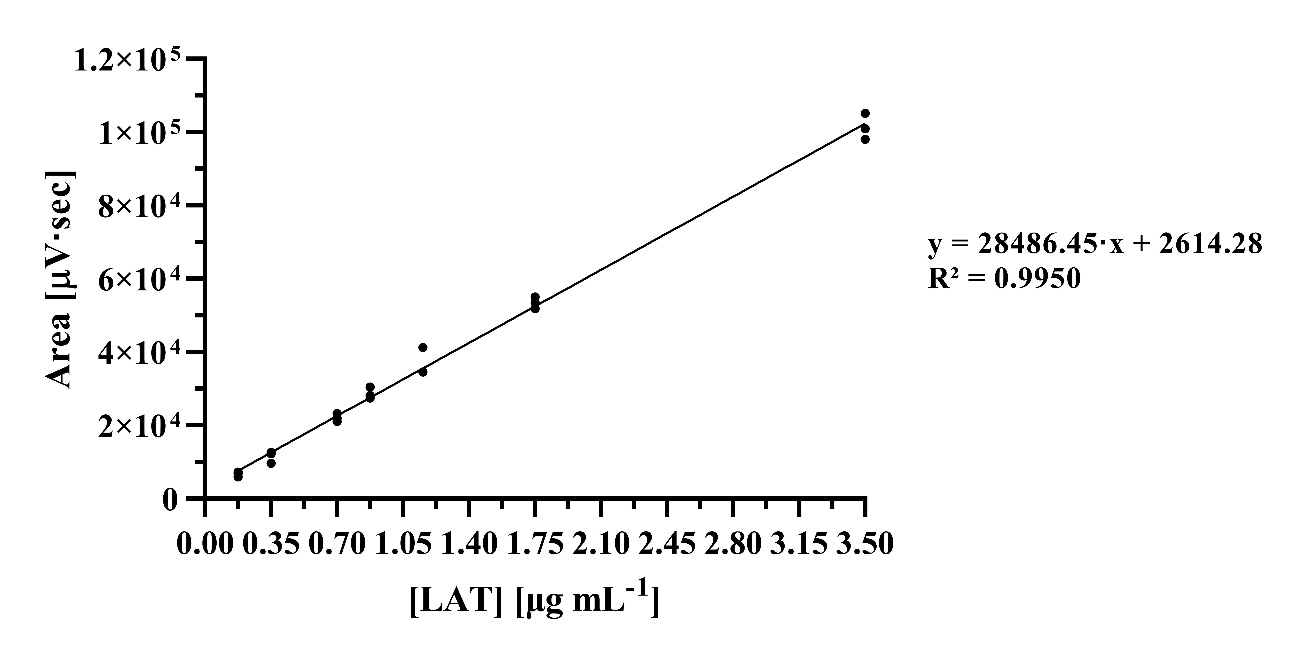


*Figure 3S. Regression line and corresponding equation used to determine LAT concentration in RTF.*
